# A Novel Role for Tm7sf2 Gene in Regulating TNFα Expression

**DOI:** 10.1371/journal.pone.0068017

**Published:** 2013-07-23

**Authors:** Ilaria Bellezza, Rita Roberti, Leonardo Gatticchi, Rachele Del Sordo, Maria Grazia Rambotti, Maria Cristina Marchetti, Angelo Sidoni, Alba Minelli

**Affiliations:** 1 Dipartimento di Medicina Sperimentale e Scienze Biochimiche, Sezione di Biochimica Cellulare, Università di Perugia, Perugia, Italia; 2 Dipartimento di Medicina Interna, Sezione di Biochimica, Università di Perugia, Perugia, Italia; 3 Dipartimento di Medicina Sperimentale e Scienze Biochimiche, Sezione di Anatomia ed Istologia Patologica, Università di Perugia, Perugia, Italia; 4 Dipartimento di Medicina Sperimentale e Scienze Biochimiche, Sezione di Anatomia, Università di Perugia, Perugia, Italia; 5 Dipartimento di Medicina Clinica e Sperimentale, Sezione di Farmacologia, Tossicologia e Chemioterapia, Università di Perugia, Perugia, Italia; National Institute of Environmental Health Sciences, United States of America

## Abstract

We have explored the role of Tm7sf2 gene, which codifies for 3β-hydroxysterol Δ14-reductase, an endoplasmic reticulum resident protein, in the sensitivity to endoplasmic reticulum stress and in the resulting inflammatory response. We used mouse embryonic fibroblasts, derived from Tm7sf2^+/+^ and Tm7sf2^−/−^ mice, to determine the *in vitro* effects of thapsigargin on NF-κB activation. Our results show that the Tm7sf2 gene controls the launch of the unfolded protein response and presides an anti-inflammatory loop thus its absence correlates with NF-κB activation and TNFα up-regulation. Our data also show that Tm7sf2 gene regulates liver X receptor activation and its absence inhibits LXR signalling. By expressing the hTm7sf2 gene in KO MEFs and observing a reduced NF-κB activation, we have confirmed that Tm7sf2 gene is linked to NF-κB activation. Finally we used genetically modified mice in an *in vivo* model of ER stress and of inflammation. Our results show a significant increase in renal TNFα expression after tunicamycin exposure and in the oedematogenic response in Tm7sf2^−/−^ mice. In conclusion, we have shown that the Tm7sf2 gene, to date involved only in cholesterol biosynthesis, also controls an anti-inflammatory loop thereby confirming the existence of cross talk between metabolic pathways and inflammatory response.

## Introduction

Cholesterol is an extremely important biological molecule because of its dual character: a friend as an essential component of cell membranes and a precursor for the synthesis of steroid hormones, bile acids and vitamin D; a foe as a predisposing factor for various diseases [1]. To prevent over-accumulation and abnormal deposition within the body, synthesis, partially residing in the endoplasmic reticulum (ER), and utilization of cholesterol are tightly regulated processes. The sterol regulatory element (SRE)/SRE-like sequences have been identified in the promoter region of many genes encoding for several enzymes in cholesterol biosynthesis [2]–[3]. The ER enzyme 3β-hydroxysterol Δ14-reductase (C14SR, EC 1.3.1.70), encoded by the Tm7sf2 gene, reduces the C14–C15 of unsaturated sterol intermediates [4]. As well as a role in cholesterol biosynthesis, ER has several other functions and disruption of any of these causes ER stress and activates the unfolded protein response (UPR). UPR, an important signalling pathway evolved in the ER to cope with stress, includes an increase in the folding capacity of the ER through the induction of ER resident molecular chaperones and protein foldases, a decrease in the folding demand on the ER by up-regulation of ER associated degradation (ERAD), an attenuation of general translation, and a stimulation of ER synthesis to dilute the unfolded protein load. Moreover, UPR controls either inflammatory and immune responses or apoptotic programs by regulating the activity of the transcription factor NF-κB [5]–[12]. An important stress sensor of UPR is the PKR-like ER-associated kinase (PERK), which mediates the phosphorylation of the α subunit of the eukaryotic translation initiation factor eIF2α at serine 51 (S51), resulting in the inhibition of translation initiation [12]–[13]. Besides inhibiting protein biosynthesis, PERK also promotes the translation of certain mRNAs, such as the activating transcription factor 4 (ATF4) mRNA [5], [7], [14], whose downstream target genes are involved in amino acid metabolism, glutathione biosynthesis, resistance to oxidative stress and protein secretion. Loss of cyclin D1 during ER stress leads to G1-arrest and provides the cell with an opportunity to restore cell homeostasis [15]–[16]. However, prolonged ER stress may cause caspase-mediated cell death [17]. Several cellular networks link the signalling pathways that control UPR to inflammation [18]–[19]. Here we investigated whether the Tm7sf2 gene, involved in cholesterol biosynthesis, is also involved in controlling a common adaptive mechanism for cellular responses against inflammation and ER stress.

## Results

### Tm7sf2 gene has a role in the response to stress conditions

**Figure 1:**
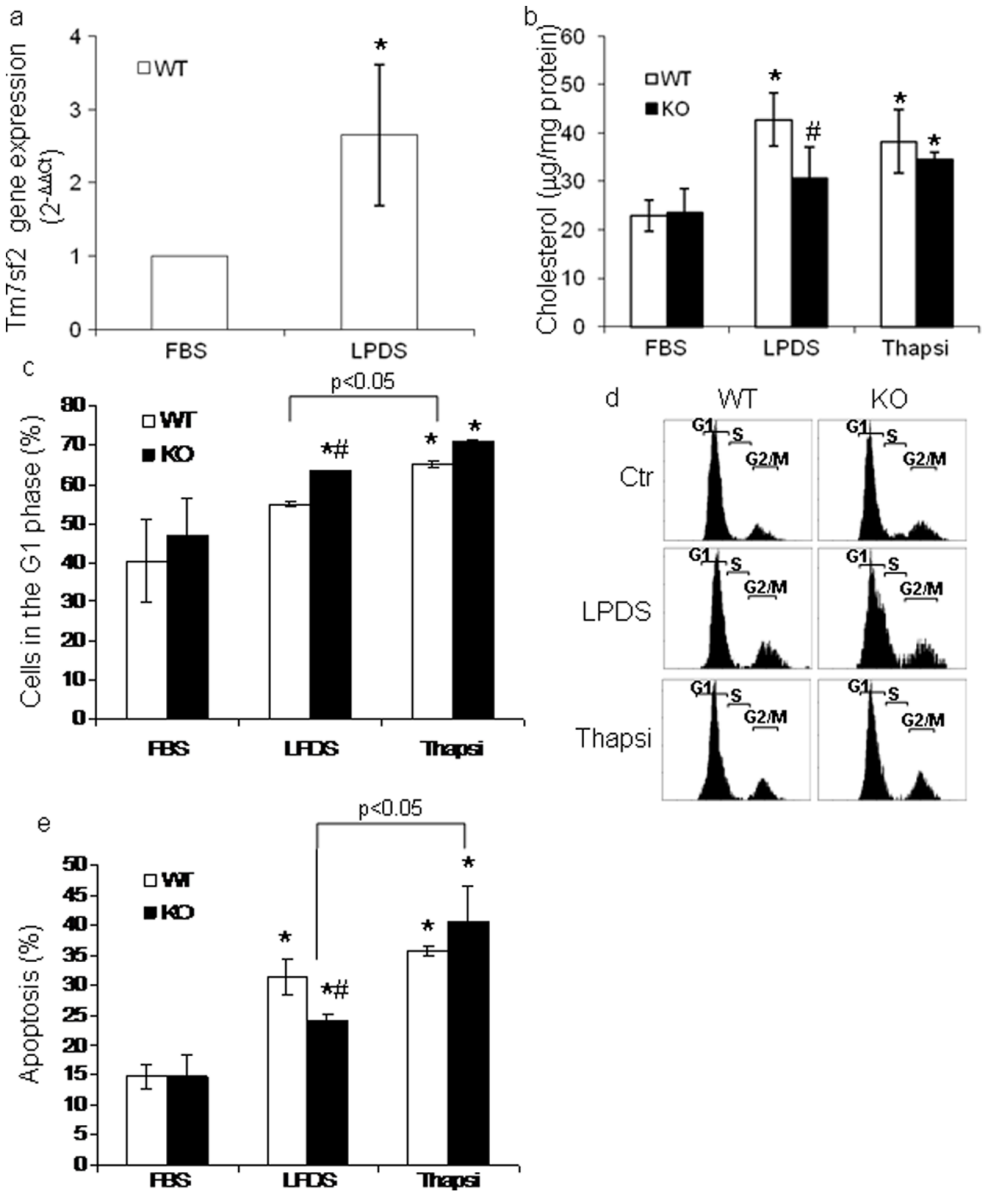
The lack of Tm7sf2 gene controls cholesterol levels and apoptotic index. (a) Expression of Tm7sf2 gene in Tm7sf2^+/+^ (WT) MEFs by real time PCR; Expression of each gene was normalized to GAPDH and reported as 2^−ΔΔCt^. Relative mRNA level of WT untreated cells was assumed as 1. (b) Cholesterol levels of WT and Tm7sf2−/− (KO) MEFs by TLC. (c) Percentage of WT and KO MEFs in the G1 phase of the cell cycle, (d) representative histogram of cell cycle distribution, (e) percentage of apoptosis by propidium iodide (PI) staining with flow cytometry. MEFs, grown in DMEM plus 5%LPDS for 24 h , were treated with 1 µM thapsigargin (Thapsi) for further 24 hr and used for analyses. Data represent mean ± s.d., (n = 5) *p<0.05 vs. FBS grown WT MEFs, # p<0.05 vs. the respective WT.

**Figure 2:**
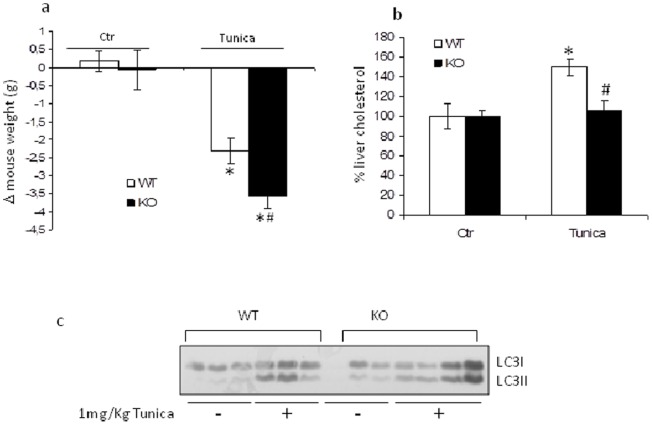
The lack of Tm7sf2 gene inhibits the increase in hepatic cholesterol levels. (a) Mouse weight changes in response to tunicamycin administration. WT and KO mice were i.p. injected with 1 mg/Kg tunicamycin and mouse weight recorded at the beginning and the end of the treatment and reported as difference between the two values for each mouse. (b) Hepatic cholesterol levels of WT and KO mice by TLC analysis. (100% = 2.45 mg/g tissue). (c) Conversion of LC3I in LC3II during TN-induced autophagy in WT and KO mice kidney by Western blotting. Data represent mean ± s.d. of n = 7 mice. *p<0.05 vs. control WT, # p<0.05 vs. the respective WT.

C14SR is an enzyme of sterol biosynthesis codified by two different genes, i.e. LBR and Tm7sf2 at 1q42 and 11q13chromosomes [23]–[24]. Functional redundancy suggests that Tm7sf2 is also involved in other physiological functions [20]. We propose that the C14SR, encoded by Tm7sf2, besides actively participating in cholesterol biosynthesis, is linked to the cellular response to ER stressors. Since cholesterol biosynthesis is tightly regulated by cholesterol levels, we first verified the induction of Tm7sf2 under sterol starving conditions. We found that Tm7sf2 gene expression was up-regulated when MEFs from WT mice were switched to lipoprotein-deficient FBS (LPDS) (Fig. 1a). Cellular cholesterol levels were also higher in WT MEFs cultured in LPDS (Fig. 1b), confirming the gene involvement in cholesterol biosynthesis. We then analyzed the effects of ER stress in MEFs of both genotypes. At basal conditions, i.e. FBS, MEFs from both genotypes showed no differences, but, when deprived of sterols, i.e. LPDS, KO MEFs showed a higher percentage of cells in the G1 phase and a reduced percentage of apoptotic cells. Exposure to thapsigargin significantly increased cholesterol levels (Fig. 1b) and the apoptotic index in KO MEFs, whereas cell-cycle distribution showed no significant differences between genotypes (Fig. 1c–e). Results, besides confirming an inverse relationship of cholesterol levels with cell cycle progression [25], clearly indicate a role for Tm7sf2 in cholesterol biosynthesis under stress conditions and in vulnerability to ER stressors. Given that thapsigargin is known to act in a sterol sensitive way [26], we then treated WT and KO mice with tunicamycin, a known ER stress inducer [27], and observed increased toxicity, as shown by weight loss during the treatment, and lower levels of hepatic cholesterol in the null genotype (Fig. 2a–b). We wondered whether the different vulnerability was accompanied by different levels of autophagy [28].

**Figure 3:**
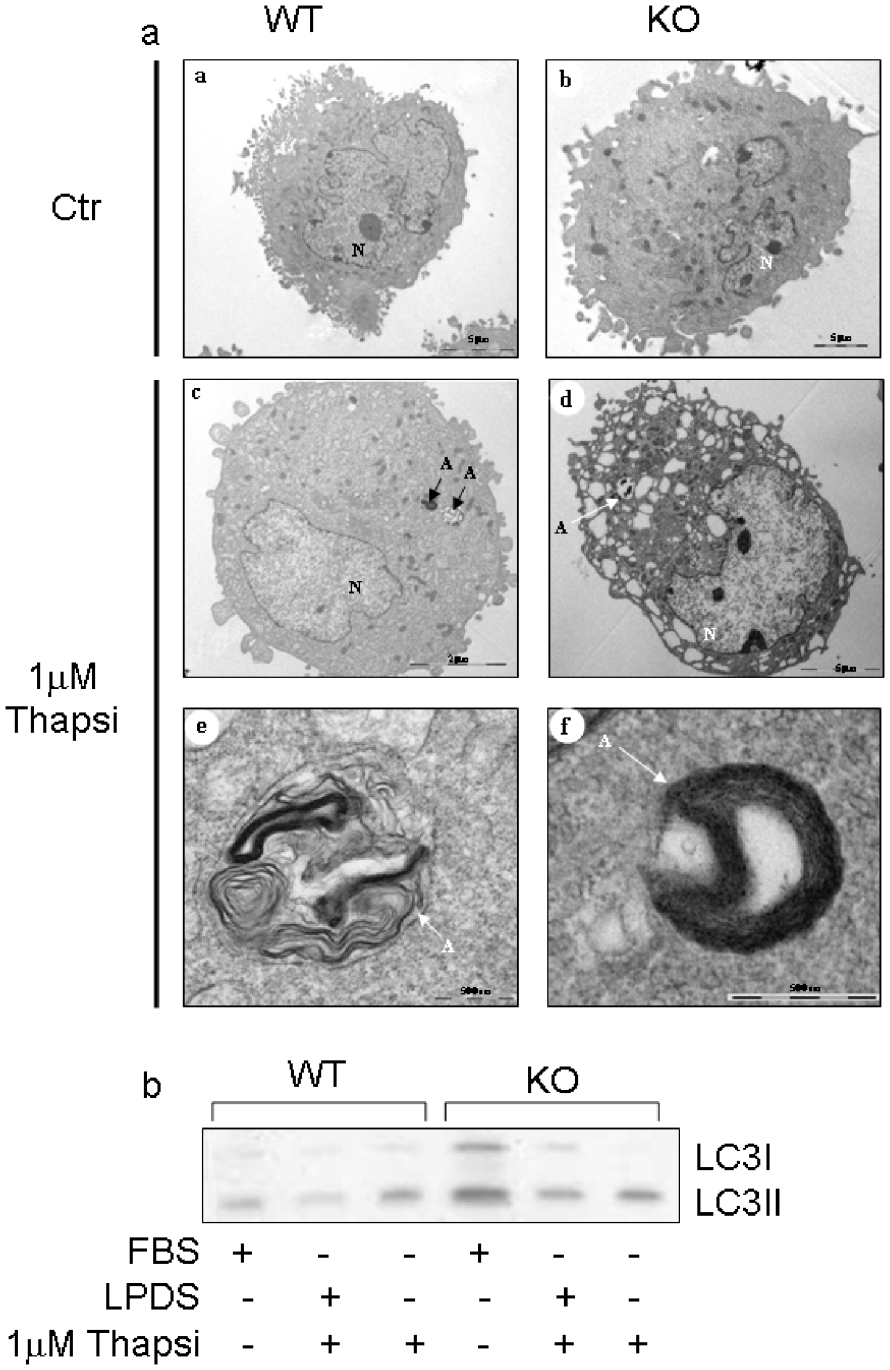
Electron microscopy analysis of autophagosome. (a) Transmission electron microscopic analysis of WT and KO MEFs. MEFs, grown in DMEM plus 5%LPDS, were treated with 1 µM thapsigargin for 24 hr, A: autophagosome, N: nucleus; (b) Conversion of LC3I in LC3II during ER stress-induced autophagy by Western blotting.

Monitoring the autophagic activity in the kidneys from both genotypes, we observed no differences in the conversion of the LC3I to LC3II form (Fig. 2c and S2). Moreover, we found that MEFs showed autophagosome formation (Fig. 3a) as well as the conversion of the LC3 I to LC3 II form (Fig. 3b and S2) in both genotypes, suggesting that autophagic activity is independent of the Tm7sf2 gene and that thapsigargyn is not responsible for blocking autophagy [29].

### Tm7sf2 gene controls eIF2α activation and attenuates ATF4 protein levels

**Figure 4:**
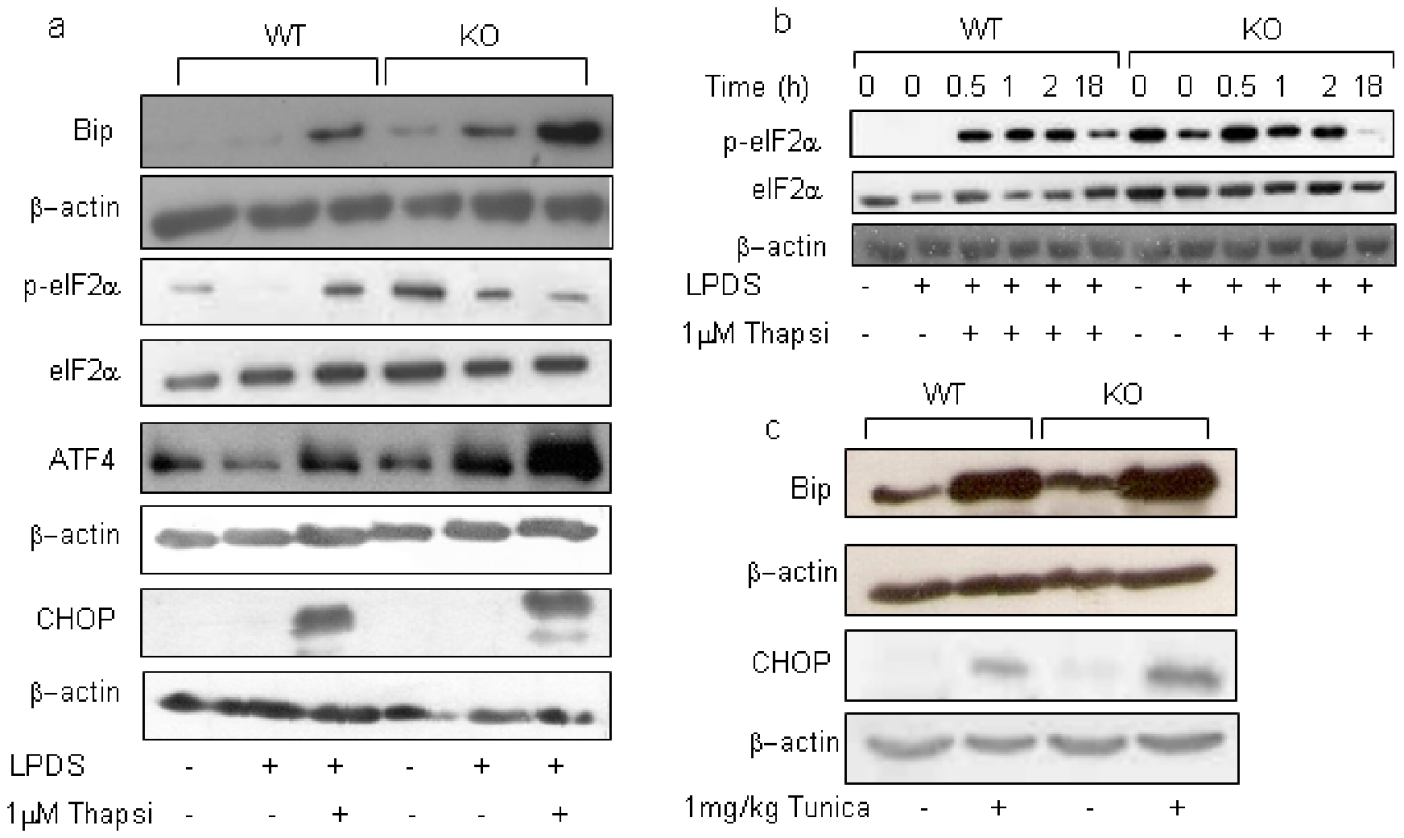
Tm7sf2 gene controls eIF2α activation and attenuates ATF4 protein levels. (a) ER stress response in WT and KO MEFs. MEFs, grown in DMEM plus 5%LPDS, were treated with 1 µM thapsigargin for 18 hr, lysed and analyzed by Western blotting with the indicated antibody. β-actin and eIF2α were used as loading controls. (b) Time-course of eIF2α phosphorylation. WT and KO MEFs, treated for the indicated time with 1 µM thapsigargin, were lysed and analyzed for eIF2α phosphorylation. β-actin and eIF2α were used as loading controls. (c) ER stress response activation in mice. Kidneys of WT and KO mice, i.p. injected with 1 mg/Kg tunicamycin and sacrificed at 48 hr, were analyzed by Western blotting with the indicated antibody. β-actin was used as loading control.

UPR network, whose initial goal is to adapt the cells to the changing environment and re-establish normal ER function, is modulated by the 78-kDa glucose-regulated protein/immunoglobulin binding protein (GRP78/Bip) [5]. When unfolded proteins accumulate, Bip dissociates from the ER stress sensor proteins and the UPR is launched via their activation. To further investigate the role of Tm7sf2 in the response to specific UPR inducers, we used MEFs from both genotypes and determined the expression of ER stress sensors (Fig. 4a and S1). Under basal conditions, Bip levels were higher in KO MEFs and, after a 18 hr thapsigargin exposure, were still markedly higher in KO MEFs. PERK activation leads to eIF2α phosphorylation which in turn attenuates total protein synthesis while promoting the translation of specific mRNAs, i.e. ATF4 mRNA [7], [30]. Following thapsigargin treatment, high p-eIF2α levels were observed in WT MEFs while ATF4 was markedly increased in KO MEFs. eIF2α phosphorylation is a key step in maintaining a balance between life and death [12]. Given the high ATF4 levels and the reduced p-eIF2α levels observed in KO MEFs, we performed a time–course analysis of the eIF2α phosphorylation (Fig. 4b). We found that, at basal and sterol-deprived conditions, i.e. FBS and LPDS, KO MEFs showed high p-eIF2α levels, suggesting that the lack of Tm7sf2 by itself causes eIF2α activation. Moreover, in WT MEFs phosphorylation peaked at 30 min and was still present at 18 hr while in the KO MEFs phosphorylation was not visible at 18 hr. Activation of ER stress and subsequent ER stress–induced apoptosis is mainly mediated by CCAAT/enhancer binding protein (C/EBP) homologous protein (CHOP), which is downstream of the PERK–eIF2α–ATF4 pathway [31]–[32].

Exposure to thapsigargin resulted in a marked increase in CHOP protein levels independently of the genetic background (Fig. 4a). We also observed that kidney of KO mice, showing higher Bip protein levels, had slightly increased expression of Bip and CHOP protein levels after a 48 hr tunicamycin exposure, which confirmed the role of the Tm7sf2 gene in the response to ER stressors (Fig. 4c).

### Tm7sf2 gene presides over an anti-inflammatory loop

**Figure 5:**
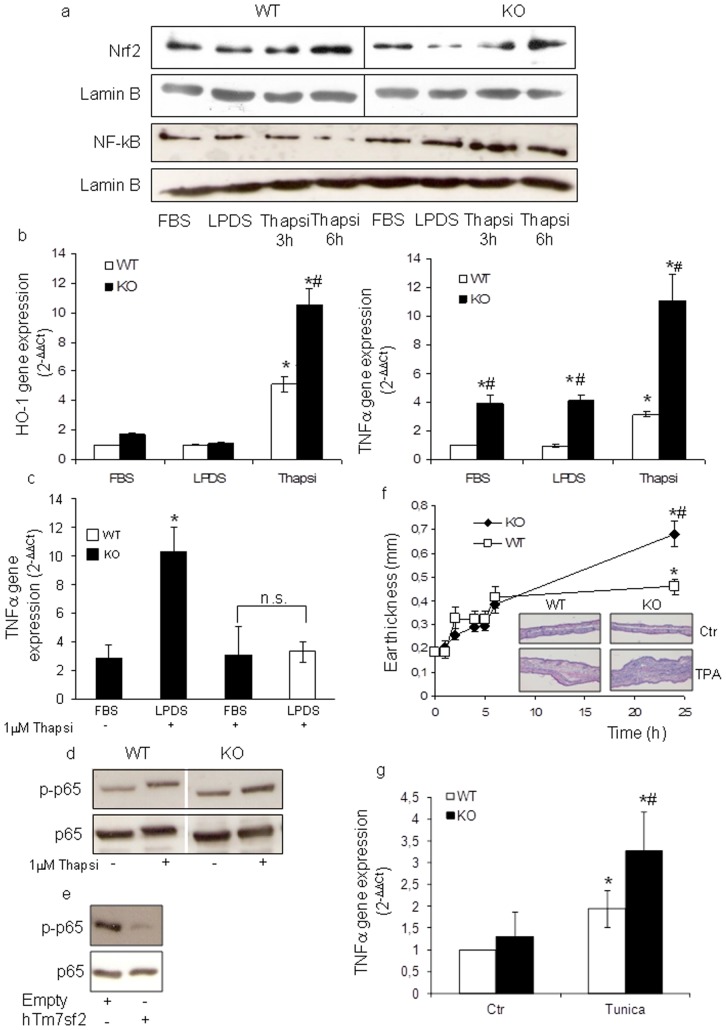
Tm7sf2 gene presides over an anti inflammatory loop. (a) Nuclear translocation of Nrf2 and NF-κB in WT and KO MEFs. MEFs, grown in DMEM plus 5%LPDS, were treated with 1 µM thapsigargin. At the indicated times, cells were collected and nuclear extracts subjected to Western blotting analyses with the indicated antibodies. Lamin B was used as loading control; (b) TNFα and HO-1 gene expression in WT and KO MEFs. MEFs, grown in DMEM plus 5%LPDS, were treated with 1 µM thapsigargin for 6 hr and subjected to real time PCR analysis. (c) TNFα expression in KO MEFs grown in 10%FBS and treated with 1 µM thapsigargin for 6 hr and subjected to real time PCR analysis. Expression of each gene was normalized to GAPDH and reported as 2^−ΔΔCt^. Relative mRNA level of WT untreated cells was assumed as 1. Results are given as mean ± s.d., (n = 7). *p<0.05 vs. FBS grown WT MEFs, # p<0.05 vs. the respective WT; NF-κB activation by Western blotting in (d) MEFs, grown in DMEM plus 10%FBS and treated with 1 µM thapsigargin for 6 hr, and (e) KO MEFs transfected with hTm7sf2 gene, incubated for 24 hr in culture medium containing 5% LPDS, and treated with 1 µM thapsigargin for 6 hr (n = 3); (f) Ear oedema induction in WT and KO mice. Mice were treated with 4 nmol TPA on both sides of the left ear. Ear thickness was measured at the indicated time points with a digital calliper. Values represent mean ± s.d. (n = 8). *p<0.05 vs. control WT, # p<0.05 vs. the respective WT. Representative histological sections of ear pinnae 24 hr after ear oedema induction. The sections were stained with H-E. Images magnification, ×100.(g) TNFα gene expression in WT and KO mice. Mice, i.p. injected with 1 mg/Kg tunicamycin, were sacrificed at 48 hr and kidneys used for real time PCR analysis. Expression of the gene was normalized to GAPDH and reported as 2^−ΔΔCt^ . Relative mRNA level of WT untreated mice kidney was assumed as 1. Results are given as mean ± s.d., (n = 7). *p<0.05 vs. control WT, # p<0.05 vs. the respective WT.

Cross talk of many cellular pathways provides a multi-tiered, integrated response to chemical stresses and affects survival and/or cell fate [33]–[35]. ATF4 and NF-κB interact via a regulatory feedback loop [36] and an Nrf2-dependent mechanism transcriptionally regulates ATF4 expression [37]. Thus, we decided to investigate whether and how the Tm7sf2 gene is involved in the interplay of the NF-κB and Nrf2 systems. Exposure of MEFs from KO genotype to thapsigargin resulted in a marked Nrf2 and NF-κB nuclear translocation/activation (Fig. 5a and S3) and up-regulation of HO-1 and TNFα mRNA levels (Fig. 5b). Results show that the lack of the Tm7sf2 gene interferes with the feedback loop to inflammatory stimuli. To confirm the pivotal role of Tm7sf2 induction in NF-κB activation, we determined NF-κB activation. We found that in basal conditions, i.e. FBS, thapsigargin effect was independent of the genotype (Fig. 5d) and that TNFα expression in thapsigargin-treated KO MEFs in FBS was comparable to thapsigargin-treated WT MEFs grown in LPDS (Fig. 5c). Moreover, we reconstituted Tm7sf2^−/−^ MEF with hTM7SF2 24 hr before thapsigargin treatment and found that hTM7SF2 expression reversed NF-κB activation (Fig. 5e and S3).To confirm in vivo the relationship between inflammatory genotype and lack of the Tm7sf2 gene, we subjected mice to a model of acute skin oedema induced by a single topical application of TPA on the ear. A time-dependent induction of the oedematogenic response was observed in TPA-treated mice. The increase in ear oedema was first detected after 6 hr of TPA treatment, remaining elevated for at least 24 hr with KO mice showing a significant increase in the oedematogenic response (Fig. 5f). On hematoxylin & eosin-stained sections, TPA application produced a marked increase in ear thickness and an abundance of inflammatory cells infiltrating the epidermis and dermis in KO mice. In tunicamycin-treated mice we found a significant higher renal TNFα expression, confirming the inflammatory genotype in KO mice (Fig. 5g).

### Tm7sf2 gene regulates LXR activation

**Figure 6:**
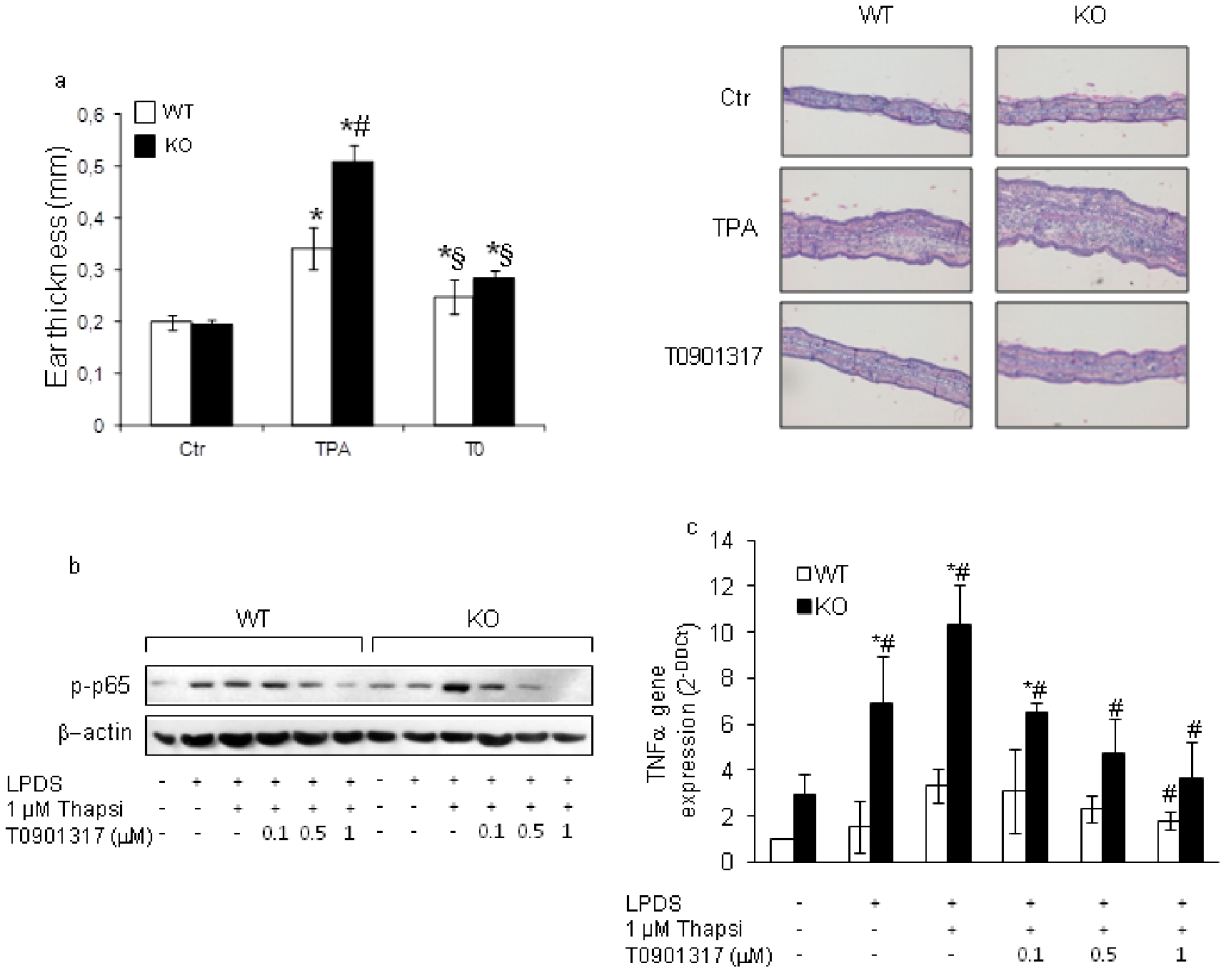
Tm7sf2 gene regulates LXR activation. (a) Ear oedema induction in WT and KO mice in the presence of T0901317. Mice were treated with 4 nmol TPA on both sides of the left ear and with 10 mM T0901317 at 45 min and 4 hr after TPA application. Ear thickness was measured after 24 hr with a digital calliper. Values represent mean ± s.d., (n = 8). *p<0.05 vs. control WT, # p<0.05 vs. the respective WT. § p<0.05 vs. the respective TPA-treated ear. Representative histological sections of ear pinnae 24 hr after ear oedema induction. The sections were stained with H-E. Images magnification, ×100. (b) NF-κB activation and (c) TNFα expression in MEFs grown in DMEM plus 5% LPDS, pre-treated for 1 hr with increasing concentrations of T0901317, then treated with 1 µM thapsigargin for 6 hr, and subjected to Western blotting and real time PCR analyses. Expression of each gene was normalized to GAPDH and reported as 2^−ΔΔCt^. Relative mRNA level of WT untreated cells was assumed as 1. Results are given as mean ± s.d., (n  = 4). *p<0.05 vs. FBS grown WT MEFs, # p<0.05 vs. the respective WT.

Activation of Liver X receptors (LXRs) negatively regulates inflammatory gene expression via blockade of NF-κB signalling [38]–[42]. To investigate whether the lack of the Tm7sf2 gene results in a decreased activation of LXR, we subjected mice of both genotypes to TPA-induced ear acute skin oedema in the presence of T0901317, a synthetic agonist of the LXRs (EC_50_∼50 nM). The increase in ear oedema, ear thickness and abundance of inflammatory cells infiltrating the epidermis and dermis were significantly reduced in both genotypes (Fig. 6a) by the activation of LXRs (Fig. S4). We also treated MEFs from both genotypes with thapsigargin in the presence of increasing T0901317 concentrations. We found that in KO MEFs the agonist induced repressive effects on NF-κB activation and TNFα expression with a similar concentration-dependent pattern. The agonist, at each concentration, caused a noticeable decrease in NF-κB activation and TNFα expression whereas in WT MEFs it caused a significant effect only at 1 µM, indicating a different sensitivity of the receptor to the agonist in the two genotypes (Fig. 6b–c). The up-regulation of ABCA1 expression confirmed the involvement of LXR activation (Fig. S4).

## Discussion

Results of this study show that the Tm7sf2 gene presides over an anti-inflammatory loop and its absence correlates with an inflammatory phenotype, i.e. NF-κB activation and TNFα up-regulation. The protein encoded by the Tm7sf2 gene resides in the ER [24], thereby we speculated that the lack of this protein/enzyme would impair ER physiological functions. Disturbance in the normal functions of the ER leads to the UPR, an evolutionarily conserved cellular stress response, initially aimed at compensating for damage. However, when ER dysfunction is severe/prolonged, it eventually triggers cell death by activating NF-κB and initiating alarm signalling pathways [8]. The NF-κB system generates the signals required for Nrf2 activation, an emergency system orchestrating cellular responses to environmental and endogenous stress. Activation of the Nrf2 system, finalized to dampen oxidative responses to inflammatory stimuli, occurs via a concerted cross talk between phosphorylation and/or protease cascades [33]–[35]. Although intracellular signal transduction pathways may offer a confusing picture since every regulatory factor appears to be regulated by all signalling pathways and to regulate all cell processes [43], our use of appropriate models renders the observed networks of regulation highly Tm7sf2-specific.

We first observed that KO MEFs, when exposed to ER stressors such as thapsigargin and tunicamycin, responded in a different manner, with thapsigargin being more effective than tunicamycin (data not shown). Considering the different mechanism of action of these two ER stress-inducers, tunicamycin, an N-glycosylation inhibitor, and thapsigargin, an ER Ca^2+^-ATPase inhibitor, it is probable that, *in vitro*, the KO genotype is more susceptible to ER stress stimuli which cause a protein accumulation, than to ER stress stimuli which perturb Ca^2+^ homeostasis. Therefore we selected thapsigargin for further *in vitro* analysis. Then, we showed that, in the absence of the Tm7sf2 gene, MEFs exposed to thapsigargin responded with increased NF-κB and Nrf2 nuclear translocation which correlates with up-regulation of HO-1 and TNFα genes, and that Tm7sf2^−/−^ mice respond with an increased oedematogenic response. Both Nrf2 and ATF4 are coordinately activated by oxidative stress and cooperate in the regulation of HO-1 gene expression, a key player in the development of tolerance in response to oxidant/electrophilic stress [37], [44]–[46]. Our results indicate the existence of a negative feedback loop provided by the Tm7sf2 gene that leads to a fine regulation of the expression of ATF4 as a regulator of the adaptive response to stress. Following UPR activation, PERK-dependent phosphorylation of eIF2α at S51 attenuates the translation of a majority of cellular proteins while promoting increased translation of select target proteins, including ATF4 and CHOP, which work as transcriptional factors regulating genes involved in either survival or death [12], [32], [47]–[48]. Phosphorylation of eIF2α is also central to the activation of the NF-κB pathway that protects the cells from the negative consequences of the oxidative stress [30], [36], [49]. In our study we found that Bip, the inductor of UPR, and p-eIF2α were higher in the null genotype at basal conditions, suggesting that the lack of Tm7sf2 alone alerts the cellular UPR. Similar results were observed in kidney from KO mice. Moreover, the different timing in the phosphorylation of eIF2α suggests a direct role for the Tm7sf2 gene in the translational control of UPR. Finally, we hypothesized that the reduced activation of LXRs might represent a plausible mechanism by which the lack of Tm7sf2 gene could lead to increases in TNFα expression. LXRs are ‘cholesterol sensors’ that, besides regulating the expression of genes involved in lipid/cholesterol metabolism in response to specific oxysterol ligands [37], negatively regulate inflammatory gene expression [18]–[19], [39]–[40], [50]–[52]. Thus, by showing that the two genotypes had a different sensitivity to the LXR agonist, we produced evidence that the Tm7sf2 gene is linked to immune functions.

In conclusion, although additional mechanisms can surely be involved, here we have shown that the Tm7sf2 gene, to date involved only in cholesterol biosynthesis, also controls an anti-inflammatory loop thereby confirming the existence of cross talk between metabolic pathways and inflammatory response.

## Materials and Methods

### Materials

All the reagents, unless otherwise stated, were from Sigma Aldrich (St. Luis, MO). All the antibodies, unless otherwise stated, were from Santa Cruz Biotech (Santa Cruz, CA) and listed in Table S1. Cell culture reagents were from Life Technologies (GibcoBRL, Gaithersburg, MD). Lipoprotein deficient serum (LPDS) was prepared according to Bennati et al. [4].

### Animals

C57BL/6 Tm7sf2^+/+^ and Tm7sf2^−/−^ mice [20] were housed at the Laboratory Animal Research Centre of Perugia University. The animals were maintained at a constant temperature of 24°C, 12 hr light/dark cycle, and fed ad libitum.

### Ethics Statement

All experimental procedures were carried out in accordance with European Directives, approved by the Institutional Animal Care and Use Committee of Perugia University (106/2012). Efforts were made to minimise animal stress/discomfort.

### Animal treatment

Skin inflammation was induced in the right ear of male C57BL/6, Tm7sf2^−/−^ and Tm7sf2^+/+^ mice by the topical application of 4 nmol of 12-O-tetradecanoylphorbol-13-acetate (TPA) dissolved in acetone on both side of the left ear. The right ear of each animal received the same volume of vehicle and was used as control. The ear oedema was assessed at the indicated time after TPA application and expressed as the increase in ear thickness. Ear thickness was measured by a digital calliper (PCE-DCP 200N, PCE Italia Srl , Italy) applied near the tip of the ear just distal to the cartilaginous ridges and the thickness was recorded in mm. To minimise variation due to technique, a single investigator performed the measurements throughout the experiment.

The anti-inflammatory effect of the LXRs agonist T0901317 was determined by topical application on the right ear of 10 mM/ear of T0901317 at 45 min and 4 hr after TPA application.

Male C57BL/6J, Tm7sf2^−/−^, and Tm7sf2^+/+^ mice were intraperitoneally injected with 1 mg/kg tunicamyicin [21] or vehicle (0.9% saline). Mice were sacrificed at 48 hr and organs used for analyses.

### Mouse Embryo Fibroblast (MEFs) preparation, treatment, and transfection

MEFs were prepared from individual embryos, bearing Tm7sf2^+/+^ (WT) and Tm7sf2^−/−^ (KO) genotypes, at embryonic day13.5 (E13.5). MEFs were maintained in DMEM containing 10% FBS (Lonza, Milan, Italy) and switched to LPDS 24 hr prior to treatments. C14SR was expressed in Tm7sf2^−/−^ MEFs by transfecting cells with the cDNA encoding human C14SR, subcloned in the mammalian expression vector pCMV-SPORT6 (Open Biosystems, clone MHS1010–73691). The GenJet™ in vitro DNA tranfection reagent for MEFs (SignaGen Laboratories, Rockville, MD) was used according to manufacturer's procedures. Briefly, 90–100% confluent cells were detached by trypsinization and transfection complex (0.7 µg plasmid DNA, transfection reagent∶DNA ratio 3∶1) was added to 1.2×10^6^ pelleted cells. After incubation for 20 min at 37°C, cells were seeded in 6-well plates in DMEM containing 10% FBS. After 16 hr, cells were incubated for additional 24 hr in culture medium containing 5% LPDS, then subjected to thapsigargin treatment for 6 hr.

### Cholesterol determination

Cholesterol, after lipids extraction and saponification, was separated by thin-layer chromatography and visualized with Cu-acetate reagent [22]. Images were acquired using the VersaDoc Imaging System and signals were quantified using Quantity One software (Bio-Rad, Milan, Italy). Purified cholesterol standard was run on the same plate as the samples to construct calibration curves.

### Electron Microscopy

MEFs from both genotypes were fixed in glutharaldehyde 2.5% in 0.1 M cacodylate buffer pH 7.4 prior to post-fixation in osmium tetroxide and uranylacetate en bloc staining. Samples were processed and embedded in epoxy resin, thin sectioned, and counter-stained with lead citrate. Digital images were obtained with a Phillips TEM 208 electron microscope (Electron Microscopy Centre, University of Perugia).

### Cell cycle analysis and apoptosis determination by flow cytometry

Cells, grown in FBS, were switched to LPDS for 24 hr and then exposed to 1 µM thapsigargin for a further 24 hr prior to propidium iodide (PI) (50 µg/ml in 0.1% sodium citrate plus 0.1% Triton X-100) addition. The PI fluorescence of individual nuclei was measured by flow cytometry using standard FACScan equipment (Becton Dickinson, Franklin Lakes, NJ). The data were recorded in a Hewlett Packard (HP 9000, model 310; Palo Alto, CA) computer. The percentage of apoptotic cell nuclei (subdiploid DNA peak in the DNA fluorescence histogram) and the percentage of cells in the phases of the cell cycle were calculated with specific FACScan research software (Lysis II). At least 10,000 events were analysed in each sample.

### Real-time RT-PCR

Total RNA was isolated with TRIZOL Reagent (Invitrogen Srl,Milano, Italy) according to the manufacturer's instructions and cDNA was synthesised using iScript cDNA synthesis kit (Bio-Rad Lab, Hercules, CA). Real time PCR was performed using the iCycler iQ detection system (Bio-Rad Lab, Hercules, CA) and SYBR Green chemistry. Primers, obtained from Invitrogen (Invitrogen Srl,Milano, Italy), are listed in Table S1. SYBR Green RT-PCR amplifications were carried out in a 96-well plate in a 25 µl reaction volume that contained 12,5 µl of 2× iQ™ SYBR® Green SuperMix (Bio-Rad), 400 nM forward and reverse primers, and 5 to 40 ng of cDNA. In each assay, no-template controls were included and each sample was run in triplicates. Mean of C_t_ values of the samples was compared to the untreated control sample and GAPDH used as internal control. The n-fold differential ratio was expressed as 2^−ΔΔCt.^

### Histochemical analysis

Paraffin-embedded sections from mouse ears were used for histochemical analysis. Briefly, tissue samples were fixed in 10% buffered formalin, embedded in paraffin and 4 µm tissue sections stained with hematoxylin&eosin.

### Western Blotting

Cells were lysed in boiling Laemmli sample buffer or processed with NE-PER® Nuclear and Cytoplasmic Extraction Reagents (Pierce Biotechnology, Rockford, IL) according to manufacturer's instruction. Total protein samples were electrophoresed on SDS-polyacrylamide gels and transferred to nitrocellulose membranes at 100 V for 1 hr. Membranes were probed with the indicated antibodies (Table S2), which were detected using HRP-based chemiluminescence (ECL, Pierce Biotechnology, Rockford, IL).

### Statistical analysis

All results were confirmed in at least three separate experiments and expressed as mean ± s.d. Data were analyzed for statistical significance by Student's t-test. p-values<0.05 were considered significant.

## Supporting Information

File S1 **Supporting methods. Method S1. PCR.** Real time PCR method description. **Method S2. Immunocytochemistry.** Immunocytochemistry method description.
(DOC)

Table S1 **List of primers.**
(DOC)

Table S2 **List of antibodies.**
(DOC)

Figure S1 **Related to** **Figure 4** **.** (a) ATF4 and (b) CHOP gene expression in WT and KO MEFs. MEFs, grown in DMEM plus 5%LPDS, were treated with 1 µM thapsigargin for 6 hr and subjected to real time PCR analysis. Expression of each gene was normalized to GAPDH and reported as 2^−ΔΔCt^. Relative mRNA level of WT untreated cells was assumed as 1. Results are given as mean ±s.d., (n = 5). *p<0.05 vs. control WT.
(TIF)

Figure S2 **Related to** **Figure 2** **and** **3** **.** Densitometric analysis of LC3II/LC3I in (a) kidney and (b) MEFs.
(TIF)

Figure S3 **Related to** **Figure 5** **.** (a) Immunofluorescence staining of MEFs cells by anti-Nrf2 antibody (1∶50) and DAPI after 6 hr exposure to 1 µM Thapsigargin. Transfection validation. Tm7sf2−/− MEFs were transfected with empty or hTm7sf2 containing pCMV-SPORT6 vector. (b) RT-PCR and (c) Western blotting analysis of Tm7sf2.
(TIF)

Figure S4 **Related to** **Figure 6** **.** ABCA1 gene expression by Real Time PCR. (a) WT and KO mice were treated with 4 nmol TPA on both sides of the left ear and with 10 mM T0901317 at 45 minutes and 4 hr after TPA application. Values represent mean ± s.d. (n = 8). *p<0.05 vs. control WT. (b) MEFs grown in DMEM plus 5% LPDS, pre-treated for 1 hr with increasing concentrations of T0901317, then treated with 1 µM thapsigargin for 6 hr, and subjected to real time PCR analyses. Expression of the gene was normalized to GAPDH and reported as 2^−ΔΔCt^. Relative mRNA level of WT untreated cells was assumed as 1. Results are given as mean ± s.d., (n  = 4). *p<0.05 vs. FBS grown WT MEFs, # p<0.05 vs. the respective WT.
(TIF)
