# Supplementary material for: A Novel Role for Tm7sf2 Gene in Regulating TNFα Expression
Source: PLoS One. 2013 Jul 23;8(7):e68017. doi: 10.1371/journal.pone.0068017 (PMC3720723; doi:10.1371/journal.pone.0068017)
Supplement: File S1 — Supporting methods. Method S1. PCR. Real time PCR method description. Method S2. Immunocytochemistry. Immunocytochemistry method description. (DOC) [file pone.0068017.s001.doc]

**Supporting Information**

**A novel role for Tm7sf2 gene in regulating TNFα expression**

Ilaria Bellezza^*1^, Rita Roberti^2^, Leonardo Gatticchi^2^, Rachele Del Sordo^3^, Maria Grazia Rambotti^4^, Angelo Sidoni^3^, Alba Minelli^1^

Dipartimento di Medicina Sperimentale e Scienze Biochimiche, Sezione di Biochimica Cellulare^1^, Sezione di Anatomia ed Istologia Patologica^3^, Sezione di Anatomia^4^; Dipartimento di Medicina Interna, Sezione di Biochimica^2^; Università di Perugia, Perugia, 06100; Italia

### Supporting Method S1. PCR

### Total RNA was isolated with TRIZOL Reagent (Invitrogen SRL, Milano, Italy) according to the manufacturer’s instructions. cDNA was synthesized using iScript cDNA synthesis kit (Bio-Rad Laboratories Hercules, CA) and amplified using Platinum1 Taq DNA Polymerase kit (Invitrogen Ltd., Paisley, UK) according to manufacturer’s instruction (annealing temeperarure 60°C and 20 cycles). Primers for hTm7sf2 and GAPDH are listed in Table S1. PCR reaction products were fractionated through 1% agarose gel stained with 0.5 μg/ml ethidium bromide and observed with an UV transilluminator.

### Supporting Method S2. Immunocytochemistry

MEFs, seeded on chamber slides, were treated for 6 hr to 1 μM Thapsigargin and then fixed with 4% paraformaldehyde in phosphate-buffered saline (PBS) at room temperature (RT) for 20 min. After three washes with PBS, the slides were treated with 0.2% Triton X-100 in PBS for 10 min, rinsed and incubated for 60 min at RT with 10% bovine serum albumin (BSA) in PBS. The slides were incubated overnight at 4°C with rabbit polyclonal antibody anti-Nrf2 (1:50) diluted in PBS containing 1% BSA and 0.01% Triton X-100. Control samples were incubated with nonimmune serum. After rinsing, the slides were incubated in the dark at RT with Alexa 488-anti-rabbit IgG (1:1,000) (Molecular Probes-Invitrogen SRL, Milano, Italy) for 1 h and extensively washed, and the cell nuclei were stained with 0.1 mg ml^−1^ diamino-phenylindole (Molecular Probes). Slides were washed and covered with coverslips in Vectashield mounting medium (Vector Lab, Inc., Burlingame, CA, USA), and cells were analyzed with a Nikon Eclipse Te2000-S fluorescence microscope (Nikon Instruments, Spa Calenzano, Italy) equipped with Olympus analysis cell software.
