# Supplementary material for: A Novel Role for Tm7sf2 Gene in Regulating TNFα Expression
Source: PLoS One. 2013 Jul 23;8(7):e68017. doi: 10.1371/journal.pone.0068017 (PMC3720723; doi:10.1371/journal.pone.0068017)
Supplement: Table S1 — List of primers. (DOC) [file pone.0068017.s002.doc]

**Table S1. List of primers**

| **Gene name** | **Gene symbol** | **Primer sequence (F: Forward; R: Reverse)** |
| --- | --- | --- |
| Glycerhaldeyde 3-phosphate dehydrogenase | GAPDH | F:GCCAAATTCAACGGCACAGT  R:AGATGGTGATGGGCTTCCC |
| Hemeoxygenase-1 | HO-1 | F:AGGTACACATCCAAGCCGAGAA  R:CTCTGGACACCTGACCCTTCT |
| Tumour necrosis factor α | TNFα | F:GCCCACGTCGTAGCAAACCAC  R:GGCTGGCACCACTAGTTGGTTGT |
| Mouse Transmembrane 7 superfamily member 2 | mTM7SF2 | F:GCCTCGGTTCCTTTGACTTC  R:CCATTGACCAGCCACATAGC |
| Human Transmembrane 7 superfamily member 2 | hTM7SF2 | F:GCCATCCCAACTATCTTG  R:GGTGAAGTAGAGGGAGGTAG |
| ATP-binding cassette transporter | ABCA1 | F:CCAGACGGAGCCGGAAGGGT  R:GTGCCCATGTCCTCGGGAGC |
| Activating transcription factor 4 | ATF4 | F:TCTTCCCCCTTGCCTTACG  R:TGGCGAGTGTAAGGAGCTAGAAA |
| CCAAT/enhancer-binding protein homologous protein | CHOP | F:AGAGGAAGAATCAAAAACCTTCACT  R:ACTCTGTTTCCGTTTCCTAGTTCTT |
