# Supplementary material for: A Novel Role for Tm7sf2 Gene in Regulating TNFα Expression
Source: PLoS One. 2013 Jul 23;8(7):e68017. doi: 10.1371/journal.pone.0068017 (PMC3720723; doi:10.1371/journal.pone.0068017)
Supplement: Table S2 — List of antibodies. (DOC) [file pone.0068017.s003.doc]

**Table S2. List of Antibody**

| **Antibody (clone)** | **Dilution** | **Company** |
| --- | --- | --- |
| Bip/GRP78 (40) | 1:2000 | BD Transduction Laboratory |
| p-eIF2α (ser 51) | 1:1000 | Cell Signalling |
| eIF2α (FL-315) | 1:200 | Santa Cruz Biotechnology |
| ATF4 (CREB2 C-20) | 1:200 | Santa Cruz Biotechnology |
| CHOP (GADD 153 F-168) | 1:200 | Santa Cruz Biotechnology |
| Nrf2 (T-19) | 1:200 | Santa Cruz Biotechnology |
| p-NF-κB p65 (Ser 536) | 1:1000 | Cell Signalling |
| NF-κB p65 (C20) | 1:200 | Santa Cruz Biotechnology |
| LC3 | 1:1000 | Sigma Aldrich |
| C14SR serum | 1:200 | Bennati et al., 2006 |
| Lamin B (H300) | 1:200 | Santa Cruz Biotechnology |
| β-Actin (C11) | 1:400 | Santa Cruz Biotechnology |
| Donkey-anti-rabbit IgG HRP | 1:5000 | Santa Cruz Biotechnology |
| Rabbit-anti-goat IgG HRP | 1:5000 | Santa Cruz Biotechnology |
| Goat-anti-mouse IgG HRP | 1:5000 | Santa Cruz Biotechnology |
