# Supplementary figures and images for: A Novel Role for Tm7sf2 Gene in Regulating TNFα Expression
Source: PLoS One. 2013 Jul 23;8(7):e68017. doi: 10.1371/journal.pone.0068017 (PMC3720723; doi:10.1371/journal.pone.0068017)

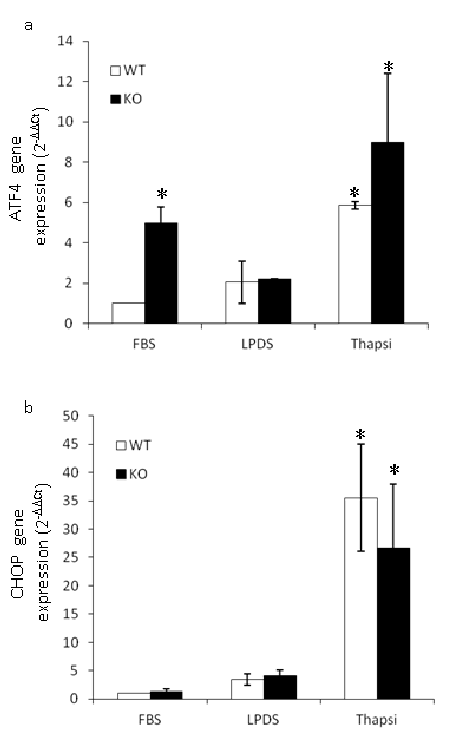

Supplement: Figure S1 — Related to Figure 4 . (a) ATF4 and (b) CHOP gene expression in WT and KO MEFs. MEFs, grown in DMEM plus 5%LPDS, were treated with 1 µM thapsigargin for 6 hr and subjected to real time PCR analysis. Expression of each gene was normalized to GAPDH and reported as 2−ΔΔCt. Relative mRNA level of WT untreated cells was assumed as 1. Results are given as mean ±s.d., (n = 5). *p<0.05 vs. control WT. (TIF) [file pone.0068017.s004.tif]

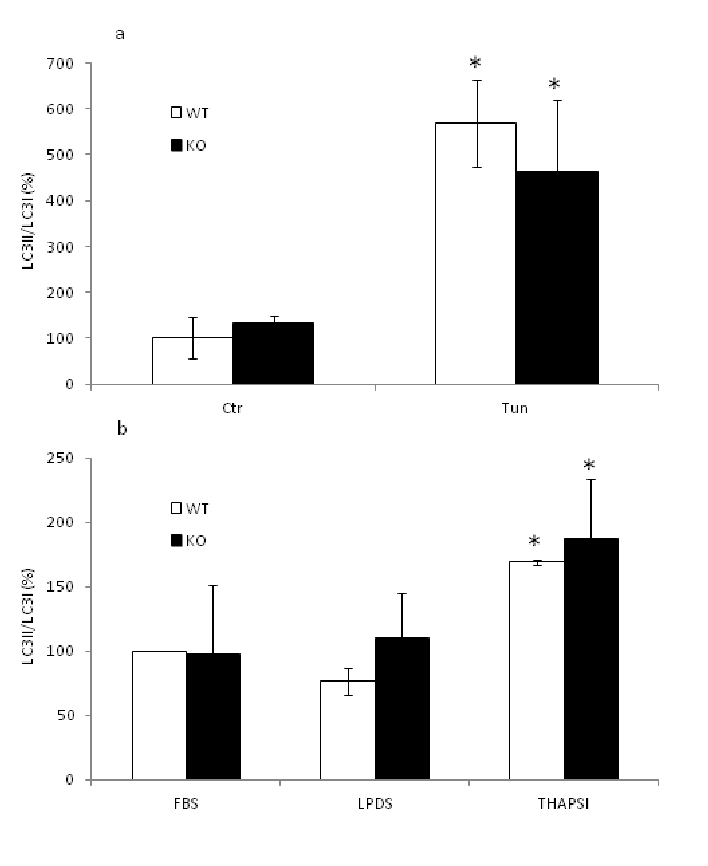

Supplement: Figure S2 — Related to Figure 2 and 3 . Densitometric analysis of LC3II/LC3I in (a) kidney and (b) MEFs. (TIF) [file pone.0068017.s005.tif]

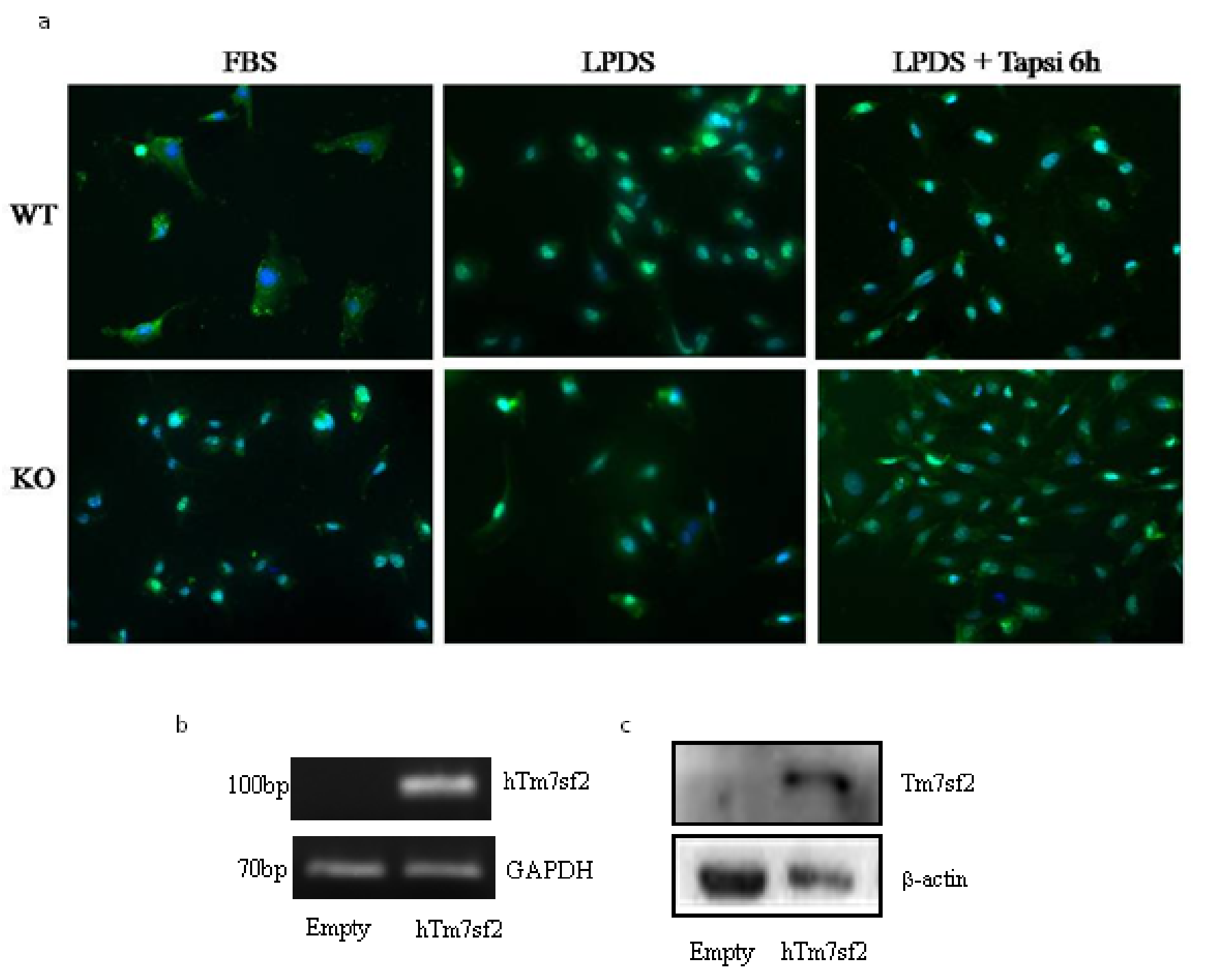

Supplement: Figure S3 — Related to Figure 5 . (a) Immunofluorescence staining of MEFs cells by anti-Nrf2 antibody (1∶50) and DAPI after 6 hr exposure to 1 µM Thapsigargin. Transfection validation. Tm7sf2−/− MEFs were transfected with empty or hTm7sf2 containing pCMV-SPORT6 vector. (b) RT-PCR and (c) Western blotting analysis of Tm7sf2. (TIF) [file pone.0068017.s006.tif]

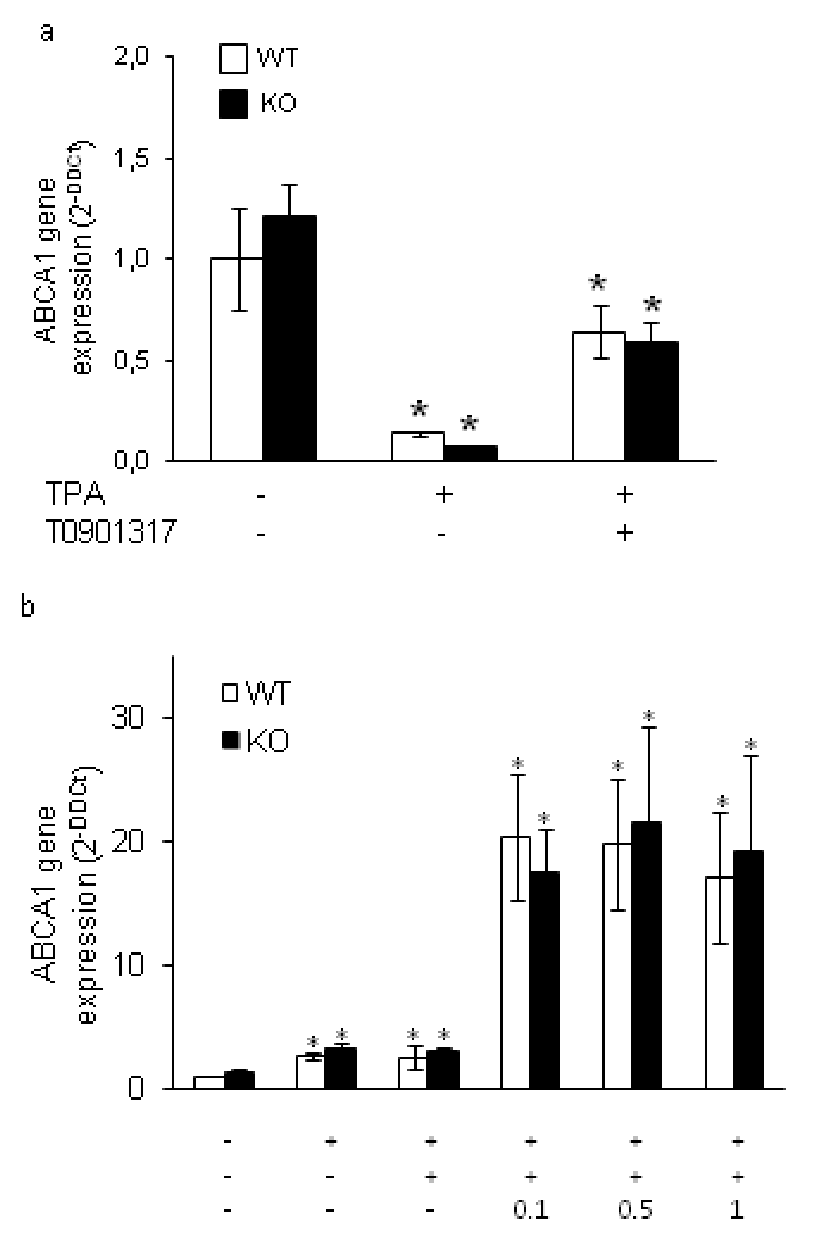

Supplement: Figure S4 — Related to Figure 6 . ABCA1 gene expression by Real Time PCR. (a) WT and KO mice were treated with 4 nmol TPA on both sides of the left ear and with 10 mM T0901317 at 45 minutes and 4 hr after TPA application. Values represent mean ± s.d. (n = 8). *p<0.05 vs. control WT. (b) MEFs grown in DMEM plus 5% LPDS, pre-treated for 1 hr with increasing concentrations of T0901317, then treated with 1 µM thapsigargin for 6 hr, and subjected to real time PCR analyses. Expression of the gene was normalized to GAPDH and reported as 2−ΔΔCt. Relative mRNA level of WT untreated cells was assumed as 1. Results are given as mean ± s.d., (n = 4). *p<0.05 vs. FBS grown WT MEFs, # p<0.05 vs. the respective WT. (TIF) [file pone.0068017.s007.tif]
